# Supplementary material for: Chloroquine resistance evolution in Plasmodium falciparum is mediated by the putative amino acid transporter AAT1
Source: Nat Microbiol. 2023 May 11;8(7):1213–26. doi: 10.1038/s41564-023-01377-z (PMC10322710; doi:10.1038/s41564-023-01377-z)
Supplement: Supplementary file 1 — Supplementary Figs. 1–7. [file 41564_2023_1377_MOESM1_ESM.pdf]

nature microbiology

Article

https://doi.org/10.1038/s41564-023-01377-z

Chloroquine resistance evolution in *Plasmodium falciparum* is mediated by the putative amino acid transporter AAT1

In the format provided by the authors and unedited

SUPPLEMENTARY FIGURES

Chloroquine resistance evolution in *Plasmodium falciparum* is mediated by the putative amino acid transporter AAT1

Alfred Amambua-Ngwa[1†], Katrina A. Button-Simons[2†], Xue Li[3†], Sudhir Kumar[4†], Katelyn Vendrely Brenneman[2†], Marco Ferrari[3], Lisa A. Checkley[2], Meseret T. Haile[4], Douglas A. Shoue[2], Marina McDew-White[3], Sarah M. Tindall[5], Ann Reyes[3], Elizabeth Delgado[3], Haley Dalhoff [2], James K. Larbalestier[2], Roberto Amato[6], Richard D. Pearson[6], Alexander B. Taylor[7], François H. Nosten[8], Umberto D'Alessandro[1], Dominic Kwiatkowski [6], Ian H. Cheeseman[9], Stefan H. I. Kappe[4,10,11], Simon V. Avery[5], David J. Conway[12], Ashley M. Vaughan[4,10]*, Michael T. Ferdig[2]*, Timothy J. C. Anderson[3]*

Supplementary Figures

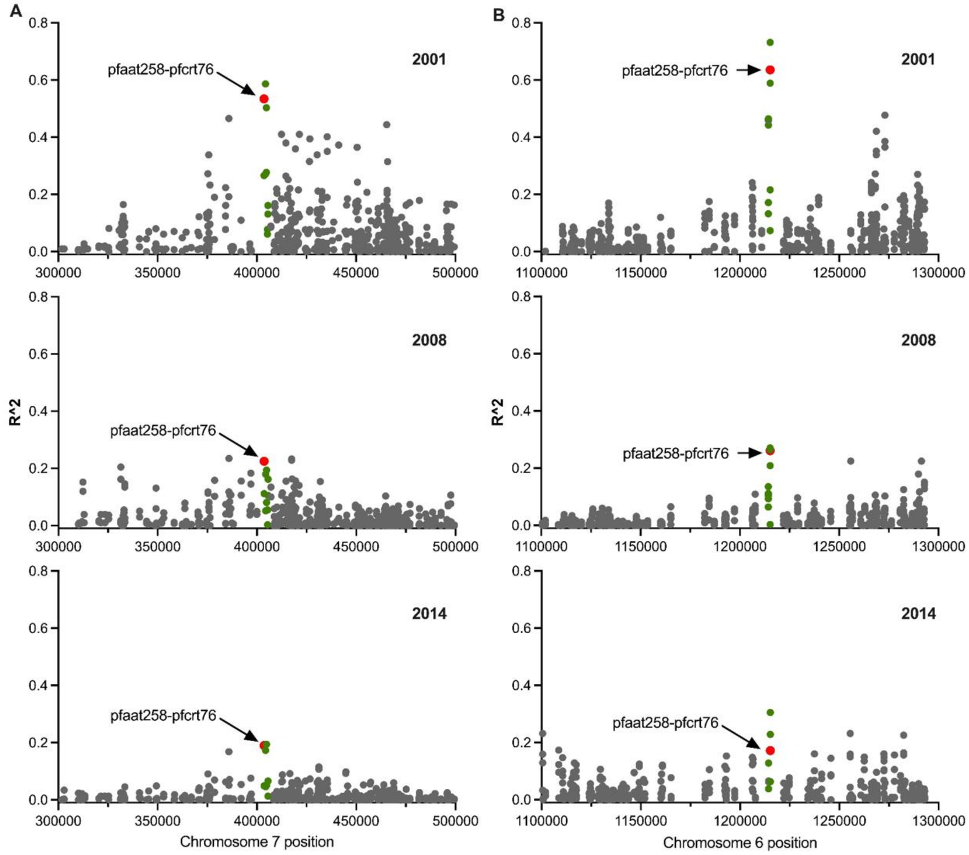

Supplementary Fig. 1. Interchromosomal linkage disequilibrium (LD) analysis. A. R^2 between *pfaat1* (SNP pfaat258 located at Pf3D7_06_v3:1215233) and a 200 kb region around the *pfcrt* gene on chr. 7. B. R^2 between *pfcrt* (SNP pfcrt76 located at Pf3D7_07_v3:403625) and a 200kb region around the *pfaat1* gene on chr. 6. R^2 values against SNPs within gene *pfcrt* (in panel A) or *pfaat1* (in panel B) are shown as green or red points, while the red points are LDs between SNPs pfaat258 and pfcrt76. For both panel A and B, from top to bottom, rows represent LD analysis for populations from year 2001, 2008, and 2014, respectively. Samples from 1984 are not shown, because neither *pfaat1* S258L nor *pfcrt* K76T were present. 1990 was omitted because only 13 samples were available. The highest LDs were observed between gene *pfaat1* and *pfcrt*.

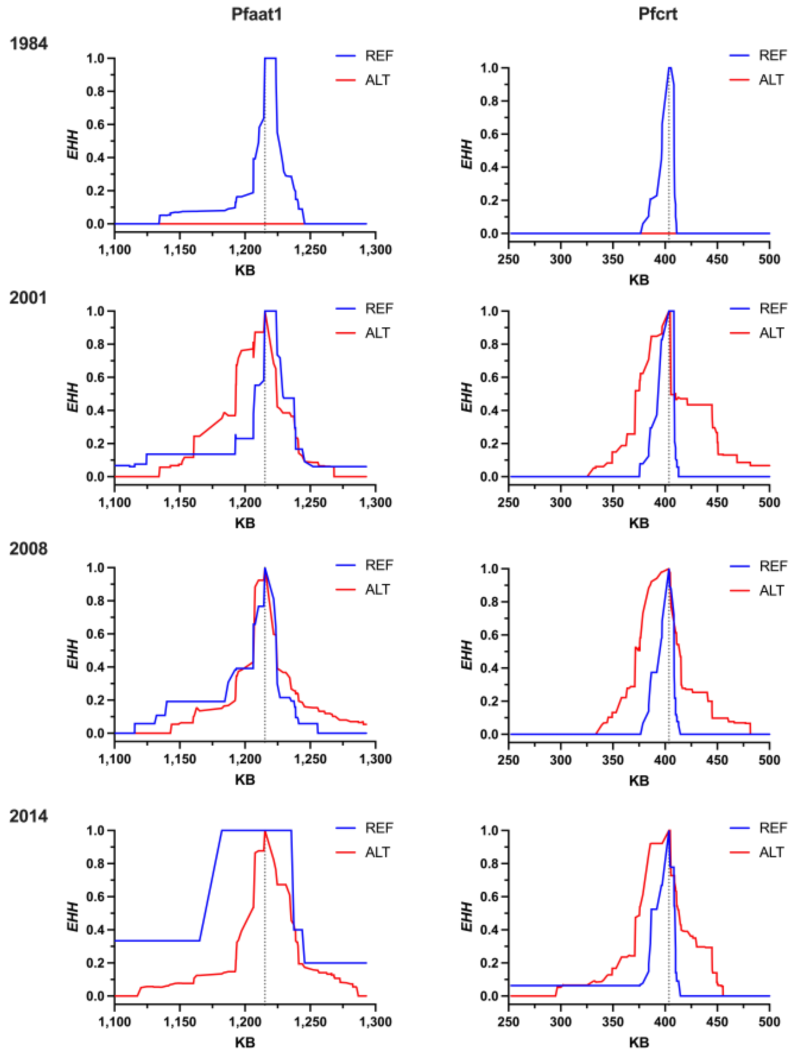

Supplementary Fig. 2. Extended haplotype homozygosity (EHH) analysis of Gambian samples. Graphs show EHH surrounding *pfaat1* S258L (left panels), or *pfcrt* K76T (right panels) in samples collected in 1984, 2001, 2008 and 2014. 1990 was omitted because only 13 samples were available. Samples from 1984 show EHH around the ancestral allele only (blue), because neither *pfaat1* S258L nor *pfcrt* K76T were sampled at that time.

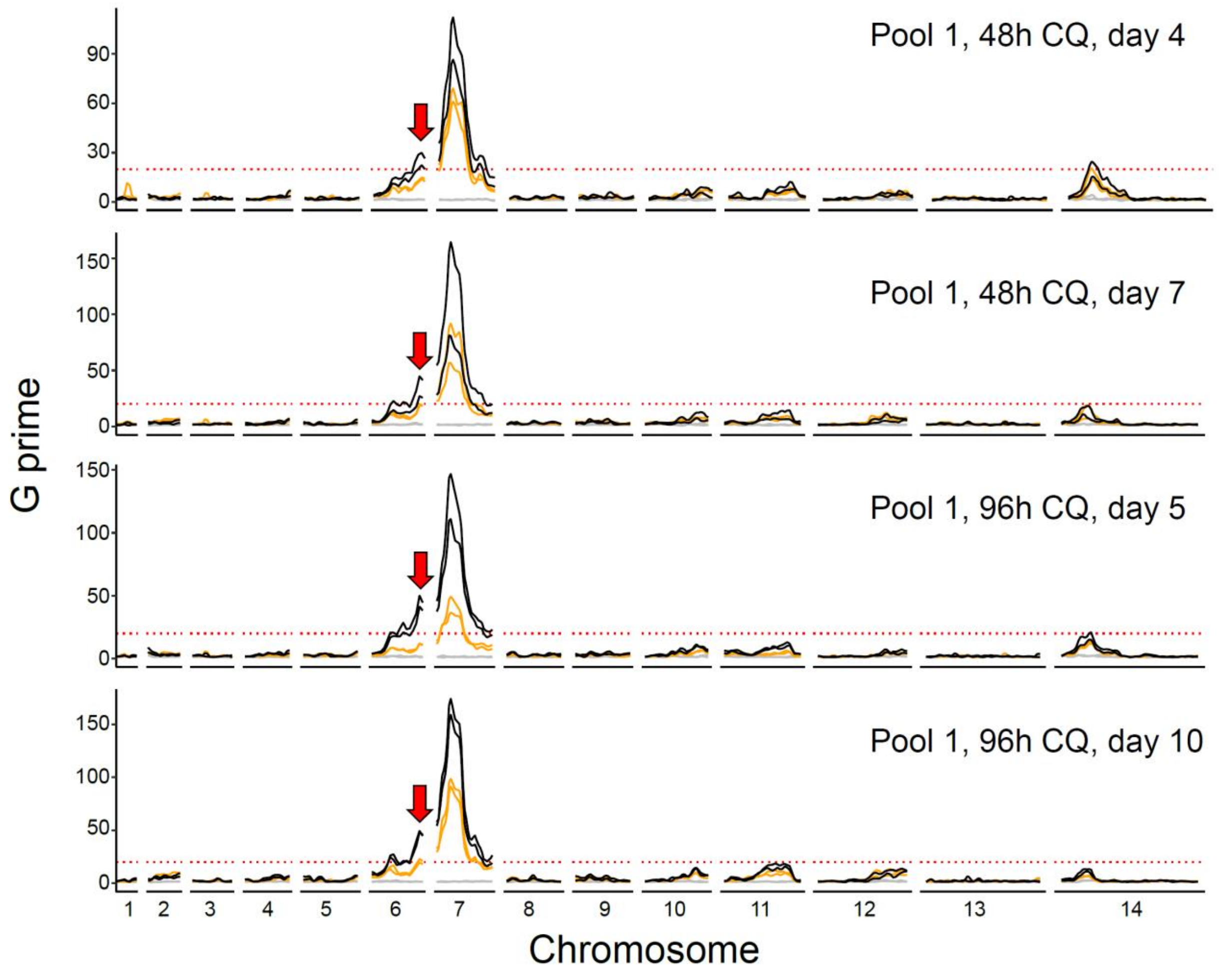

Supplementary Fig. 3. Mapping CQ resistance loci with cross 3D7×NHP4026 using recombinant progeny pool 1. Grey, orange, and black lines indicate BSAs from 50 nM, 100 nM, and 250 nM CQ treatments, separately. Lines with the same color are BSAs from technical replicates. Red dashed lines are the threshold (*G prime* = 20) for QTL detection. Red arrows indicate the location of the chromosome 6 QTL. The peak on chr 7 contains pfCRT.

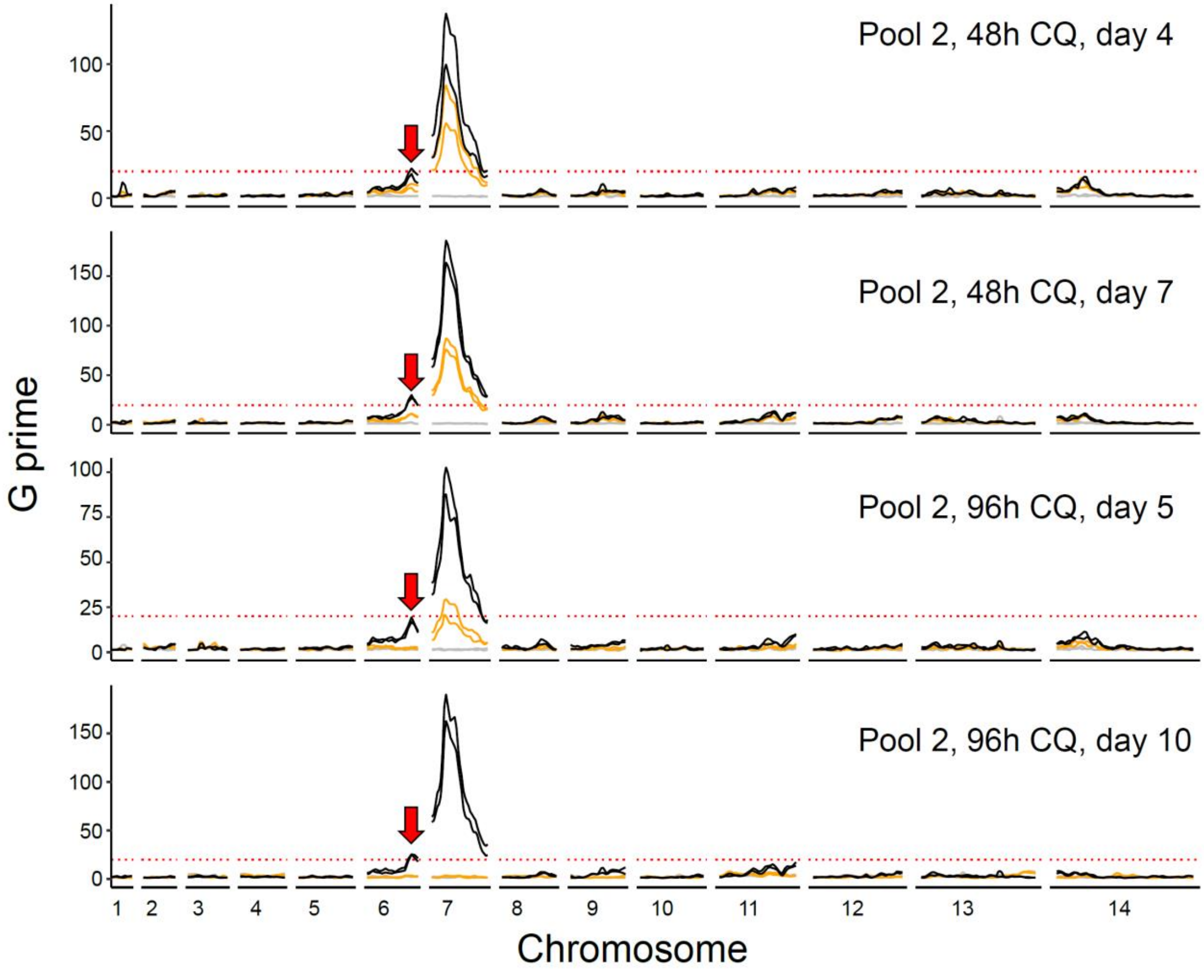

Supplementary Fig. 4. Mapping CQ resistance loci with cross 3D7×NHP4026 using recombinant progeny pool 2. Grey, orange, and black lines indicate BSAs from 50 nM, 100 nM, and 250 nM CQ treatments, separately. Lines with the same color are BSAs from technical replicates. Red dashed lines are the threshold (*G prime* = 20) for QTL detection. Red arrows indicate the location of the chromosome 6 QTL. The peak on chr 7 contains pfCRT.

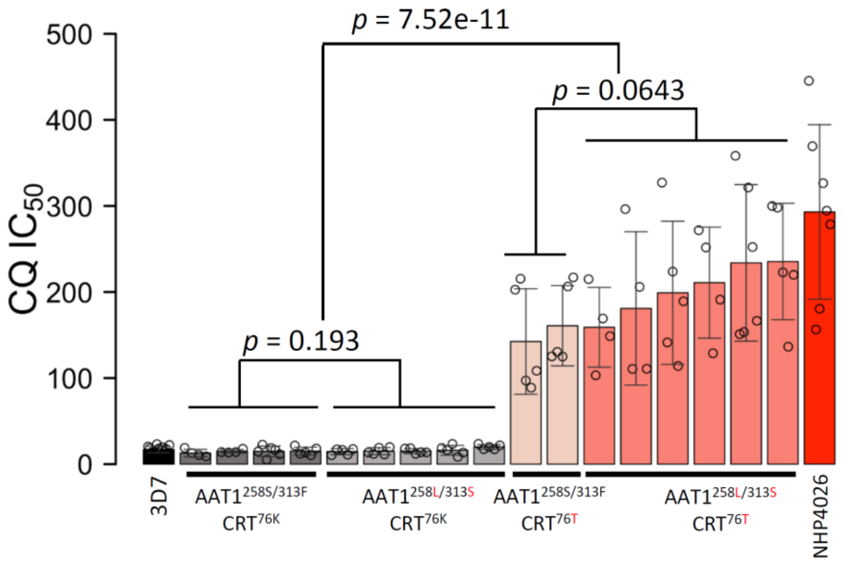

Supplementary Fig. 5. Mean IC_{50} (± S.D.) of parents and progeny grouped by combinations of *pfcrt* and *pfaat1* allele. IC_{50} for each clone is calculated from 4-13 biological replicates (Supplementary Table 8). Only two progeny carrying *pfaat1* 258S/313F (WT) and *pfcrt* K76T were recovered. *P* values indicate significance levels are based on two-way ANOVA analysis.

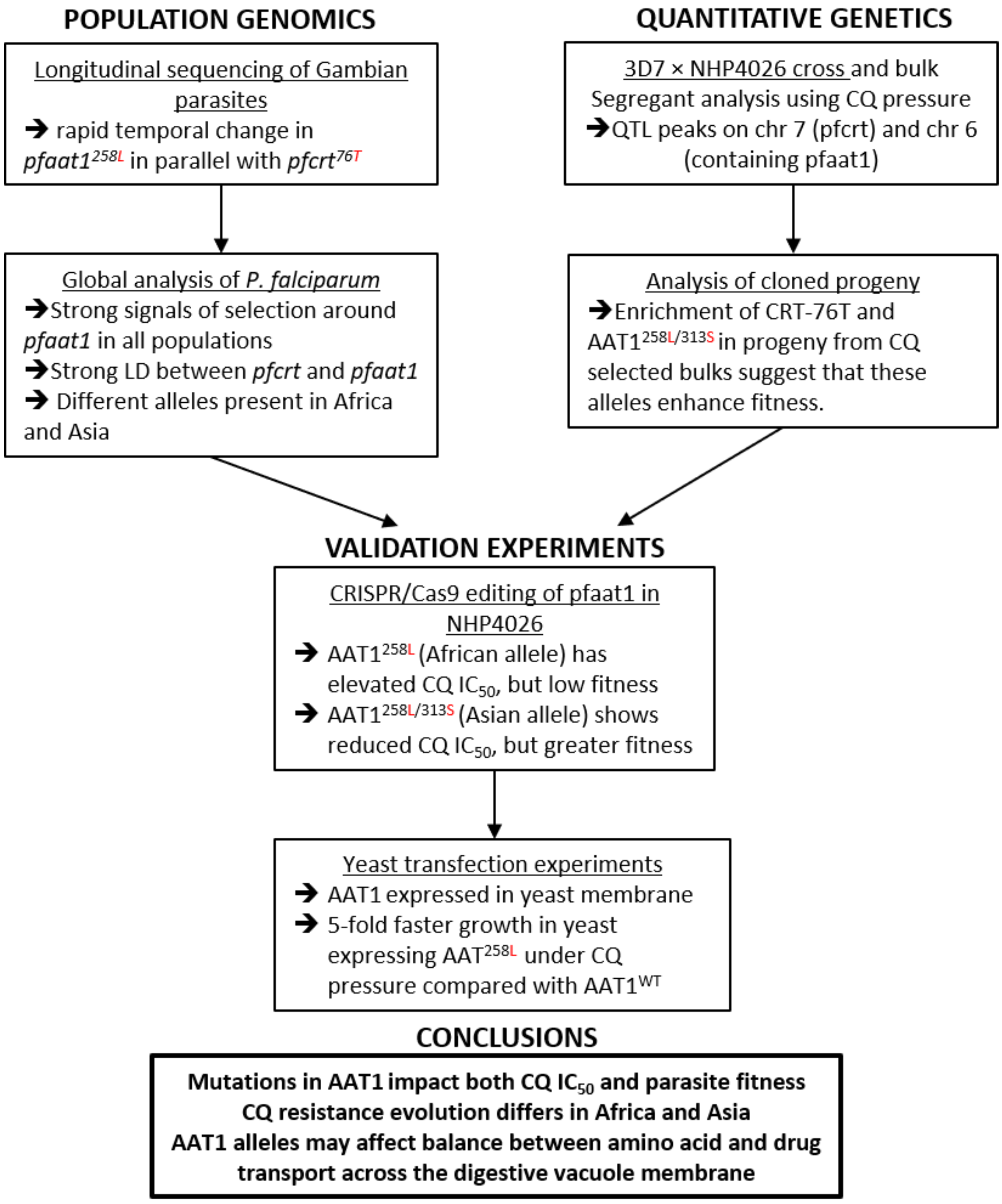

Supplementary Fig. 6. Project design. We use (i) population genomic analyses, (ii) genetic crosses and quantitative genetics analysis followed by (iii) functional analyses to investigate the role of additional loci in CQ resistance.

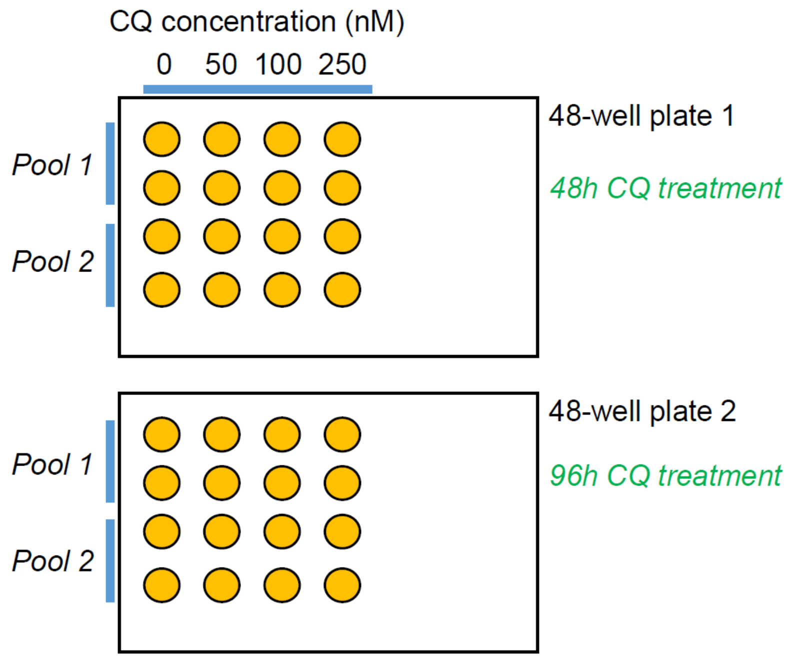

Supplementary Fig. 7. Experiment design for CQ bulk segregant analysis.
