# Supplementary material for: Chloroquine resistance evolution in Plasmodium falciparum is mediated by the putative amino acid transporter AAT1
Source: Nat Microbiol. 2023 May 11;8(7):1213–26. doi: 10.1038/s41564-023-01377-z (PMC10322710; doi:10.1038/s41564-023-01377-z)
Supplement: Supplementary file 2 — Reporting Summary [file 41564_2023_1377_MOESM2_ESM.pdf]

nature portfolio

Corresponding author(s): Ashley M. Vaughan, Michael T. Ferdig, Timothy J. C. Anderson

Last updated by author(s): Mar 25, 2023

Reporting Summary

Nature Portfolio wishes to improve the reproducibility of the work that we publish. This form provides structure for consistency and transparency in reporting. For further information on Nature Portfolio policies, see our Editorial Policies and the Editorial Policy Checklist.

Statistics

For all statistical analyses, confirm that the following items are present in the figure legend, table legend, main text, or Methods section.

n/a	Confirmed	
☐	☒	The exact sample size (*n*) for each experimental group/condition, given as a discrete number and unit of measurement
☐	☒	A statement on whether measurements were taken from distinct samples or whether the same sample was measured repeatedly
☐	☒	The statistical test(s) used AND whether they are one- or two-sided<br>*Only common tests should be described solely by name; describe more complex techniques in the Methods section.*
☐	☒	A description of all covariates tested
☐	☒	A description of any assumptions or corrections, such as tests of normality and adjustment for multiple comparisons
☐	☒	A full description of the statistical parameters including central tendency (e.g. means) or other basic estimates (e.g. regression coefficient) AND variation (e.g. standard deviation) or associated estimates of uncertainty (e.g. confidence intervals)
☐	☒	For null hypothesis testing, the test statistic (e.g. *F*, *t*, *r*) with confidence intervals, effect sizes, degrees of freedom and *P* value noted<br>*Give P values as exact values whenever suitable.*
☒	☐	For Bayesian analysis, information on the choice of priors and Markov chain Monte Carlo settings
☒	☐	For hierarchical and complex designs, identification of the appropriate level for tests and full reporting of outcomes
☒	☐	Estimates of effect sizes (e.g. Cohen's *d*, Pearson's *r*), indicating how they were calculated

Our web collection on statistics for biologists contains articles on many of the points above.

Software and code

Policy information about availability of computer code

Data collection	All raw sequencing data have been submitted to the NCBI Sequence Read Archive (SRA, https://www.ncbi.nlm.nih.gov/sra) or European Nucleotide Archive (ENA) with accession numbers available in Table S1. All other data are available in the main text or supplementary materials.
Data analysis	The code used in analysis and data analyzed are available at GitHub through the following links: https://github.com/emilyli0325/CQ.AAT1.git (XL), https://github.com/MPB-mrcg?tab=repositories (AAN), https://github.com/kbuttons/CQ.AAT1.progeny.git (KBS).

For manuscripts utilizing custom algorithms or software that are central to the research but not yet described in published literature, software must be made available to editors and reviewers. We strongly encourage code deposition in a community repository (e.g. GitHub). See the Nature Portfolio guidelines for submitting code & software for further information.

Data

Policy information about availability of data

All manuscripts must include a data availability statement. This statement should provide the following information, where applicable:

- Accession codes, unique identifiers, or web links for publicly available datasets
- A description of any restrictions on data availability
- For clinical datasets or third party data, please ensure that the statement adheres to our policy

A data availability statement is included in the manuscript
All raw sequencing data have been submitted to the NCBI Sequence Read Archive (SRA, https://www.ncbi.nlm.nih.gov/sra) or European Nucleotide Archive (ENA) with accession numbers available in Supplementary Table 1.

Human research participants

Policy information about studies involving human research participants and Sex and Gender in Research.

Reporting on sex and gender	*Use the terms sex (biological attribute) and gender (shaped by social and cultural circumstances) carefully in order to avoid confusing both terms. Indicate if findings apply to only one sex or gender; describe whether sex and gender were considered in study design whether sex and/or gender was determined based on self-reporting or assigned and methods used. Provide in the source data disaggregated sex and gender data where this information has been collected, and consent has been obtained for sharing of individual-level data; provide overall numbers in this Reporting Summary. Please state if this information has not been collected. Report sex- and gender-based analyses where performed, justify reasons for lack of sex- and gender-based analysis.*
Population characteristics	*Describe the covariate-relevant population characteristics of the human research participants (e.g. age, genotypic information, past and current diagnosis and treatment categories). If you filled out the behavioural & social sciences study design questions and have nothing to add here, write "See above."*
Recruitment	*Describe how participants were recruited. Outline any potential self-selection bias or other biases that may be present and how these are likely to impact results.*
Ethics oversight	*Identify the organization(s) that approved the study protocol.*

Note that full information on the approval of the study protocol must also be provided in the manuscript.

Field-specific reporting

Please select the one below that is the best fit for your research. If you are not sure, read the appropriate sections before making your selection.

☒ Life sciences ☐ Behavioural & social sciences ☐ Ecological, evolutionary & environmental sciences

For a reference copy of the document with all sections, see nature.com/documents/nr-reporting-summary-flat.pdf

Life sciences study design

All studies must disclose on these points even when the disclosure is negative.

Sample size	Population analysis - we used malaria samples available from longitudinal sampling of Plasmodium falciparum in the Gambia (n=315). For the global analysis we also analyzed available archived sequence data from the public databases and published manuscripts (N=4051). Power calculations were not conducted, but the numbers far exceed those needed to identify temporal or spatial trends
Data exclusions	No data were excluded from these analyses.
Replication	Replication is detailed in the manuscript. (i) We conducted two independent biological replications of genetic crosses for the linkage analysis. (ii) We used three independent CRISPR/Cas9 gene edits for each pfAAT1 haplotype generated, to avoid spurious conclusions from off target effects. (iii) We conducted multiple (10-28) independent measures of IC50 to improve robustnesss of drug response measures. (iv) We conducted 3 independent replicates of each each pairwise competition experiment when quantifying parasite fitness. Replicates provided consistent results: this is documented in the manuscript
Randomization	The bulk segregant analysis used parasite progeny that were randomly assigned to treatment or control groups - this was done by aliquoting parasite cultures for the two treatment. No randomization was conducted for the population genomic and CRISPR analyses.
Blinding	The outcome of competition experiments and Bulk segregant analyses was conducted blind, and the results were determined by genome sequencing or genotyping experiments.

Reporting for specific materials, systems and methods

We require information from authors about some types of materials, experimental systems and methods used in many studies. Here, indicate whether each material, system or method listed is relevant to your study. If you are not sure if a list item applies to your research, read the appropriate section before selecting a response.

Materials & experimental systems

n/a	Involved in the study
☒	☐ Antibodies
☐	☒ Eukaryotic cell lines
☒	☐ Palaeontology and archaeology
☐	☒ Animals and other organisms
☒	☐ Clinical data
☒	☐ Dual use research of concern

Methods

n/a	Involved in the study
☒	☐ ChIP-seq
☒	☐ Flow cytometry
☒	☐ MRI-based neuroimaging

Eukaryotic cell lines

Policy information about cell lines and Sex and Gender in Research

Cell line source(s)	No cell lines were used that were derived from human or vertebrate models
Authentication	We verified the identity of Plasmodium cultures using illumina short read sequencing
Mycoplasma contamination	The cell lines were not tested for mycoplasma contamination
Commonly misidentified lines (See ICLAC register)	None

Animals and other research organisms

Policy information about studies involving animals; ARRIVE guidelines recommended for reporting animal research, and Sex and Gender in Research

Laboratory animals	FRG NOD Huhep mice were purchased from Yecuris Corporation.
Wild animals	*Provide details on animals observed in or captured in the field; report species and age where possible. Describe how animals were caught and transported and what happened to captive animals after the study (if killed, explain why and describe method; if released, say where and when) OR state that the study did not involve wild animals.*
Reporting on sex	Male and female FRG NOD huHep mice18 with human chimeric livers were used in the experiments. Both male and female mice support liver stage P. falciparum development and can be used for these studies
Field-collected samples	*For laboratory work with field-collected samples, describe all relevant parameters such as housing, maintenance, temperature, photoperiod and end-of-experiment protocol OR state that the study did not involve samples collected from the field.*
Ethics oversight	The study was performed in accordance with the Guide for the Care and Use of Laboratory Animals of the National Institutes of Health (NIH), USA. The Seattle Children's Research Institute (SCRI) has an Assurance from the Public Health Service (PHS) through the Office of Laboratory Animal Welfare (OLAW) for work approved by its Institutional Animal Care and Use Committee (IACUC). All of the work carried out in this study was specifically reviewed and approved by the SCRI IACUC.

Note that full information on the approval of the study protocol must also be provided in the manuscript.
